# Association between butadiene exposure and leukemia mortality in synthetic rubber industry workers

**DOI:** 10.13075/ijomeh.1896.02792

**Published:** 2026

**Authors:** Lynne Pavlic Marshall, Christine R. Langton, Lindsey A. Wood

**Affiliations:** Valeo Sciences LLC, Ladera Ranch, CA, USA

**Keywords:** cancer, epidemiology, occupational exposure, risk assessment, butadiene, exposure-risk relationship

## Abstract

**Objectives:**

Regulatory agencies across the USA and EU utilize analyses of a cohort of workers in the styrene-butadiene rubber (SBR) industry to assess excess cancer risk and make policy decisions on occupational and environmental exposure limits for 1,3-butadiene (BD). The objective of this study was to perform an analysis on the SBR data, including explorations of covariates and worker inclusion criteria, to evaluate the effects on the model parameters for estimating leukemia mortality.

**Material and Methods:**

Exposure and mortality information for 21 069 male and female workers in 6 North American SBR plants were obtained. Cox proportional hazards models were used to evaluate the association between BD and leukemia mortality. All Cox models used number of days from hire date to person-day observation as the time scale and included cumulative BD as a time-varying exposure.

**Results:**

Most workers were exposed to BD (66.4%). There were 132 deaths due to leukemia. The Cox proportional hazards model excluding all workers with <1 year of employment (N = 1801) and adjusted for age, BD high intensity tasks, and year of hire resulted in a statistically significant association between cumulative BD exposure and leukemia mortality (β = 2.14 × 10^–4^, 95% CI: 0.40–3.87 × 10^–4^; HR = 1.000214, 95% CI: 1.000040–1.000387). The same model including all workers in the dataset had similar results (β = 2.21 × 10^–4^, 95% CI: 0.49–3.93 × 10^–4^; HR = 1.000221, 95% CI: 1.000049–1.000393).

**Conclusions:**

The authors' exploration of covariates and worker inclusion criteria to evaluate the quantitative effects on the relationship between BD and leukemia mortality demonstrates little variation in model parameters despite covariates and worker groups included. Using a conservative 95% upper bound of the β-coefficient to estimate excess risk resulted in a greater BD exposure estimate compared to regulatory bodies, suggesting a weaker overall exposure risk relationship.

## Highlights

- The study explored exposure-response relationship (ERR) of butadiene and leukemia mortality.
- Cox regression models were adjusted for age, peak butadiene exposure, and year of hire.
- Schoenfeld and Martingale residuals were examined to test model assumptions.
- The β estimates and standard errors showed little variation across the robust models.
- Risk estimates suggest weaker ERRs than those currently used by regulatory agencies.

## INTRODUCTION

The WHO International Agency for Research on Cancer (IARC) classified 1,3-butadiene (BD) as a group 1 carcinogen; they determined that there was sufficient evidence that BD causes hemolymphatic cancers in humans [1]. The strongest evidence of an association between BD and lymphatic cancers came from occupational cohort studies of BD monomer production workers, while the strongest evidence for an association between BD and hematological cancers and leukemia, specifically, came from occupational studies of synthetic rubber production, i.e., styrene-butadiene rubber (SBR), worker cohorts [1]. The production of SBR consists primarily of the polymerization of BD and styrene (STY), typically with a 75%, 25% composition, respectively, of the 2 monomers [2].

Excess mortality has been noted in the rubber industry since the 1920s in England and the 1940s in the USA [3]. Occupational cohorts of the SBR industry have been analyzed periodically and have shown consistent associations between BD exposure and leukemia outcomes [4–11]. Analyses of leukemia subtypes have been less consistent, possibly due to low case counts and large confidence intervals (CI). Although earlier studies included 1 or 2 SBR plants [12], more recent studies have combined USA and Canadian cohorts (the SBR cohort) to allow for more robust analyses. Mortality experience, exposure assessment, and analytical methods have been updated over time to add data and statistical power to assess exposure-response relationships (ERRs). Importantly, regarding updated analytical methods, more recent analyses of the SBR cohort shifted from Poisson regression models used in earlier studies [5,7] to Cox proportional hazard models, primarily since they allow for continuous analyses of exposure and adjustment variables such as age [4,8,9,11].

Regulatory agencies across the USA and EU (i.e., the Dutch Expert Committee on Occupational Standards [DECOS], the European Chemicals Agency [ECHA], the US Environmental Protection Agency [EPA], and the Texas Commission on Environmental Quality, [TCEQ]) use analyses of the SBR data to assess excess cancer risk and make policy decisions on occupational and environmental exposure limits for BD. More specifically, the ECHA Risk Assessment Committee (RAC) published their most recent opinion on occupational exposure limits for BD in 2024 [13]. They conducted a linear regression on grouped data (deciles of BD exposure) and used the average of 2 βs resulting from excluding the highest decile and the 2 highest deciles in their quantitative cancer risk assessment. The authors of the ECHA [13] opinion noted the limitations of modeling on grouped data, including losing specificity of individual-level exposure data, arbitrary selection of number of groups affecting β estimates, and effectively reducing sample size to the number of groups selected.

Antoniou and Kirman [14] performed a literature review and impact analysis of studies conducted on the SBR cohort through the last 2 updates of the mortality data (i.e., 1998 and 2009) in order to determine the optimal outcome, exposure, and adjustment variables that should be included in an analysis of the data for regulatory agencies to conduct cancer risk assessments. The authors evaluated all previous statistical models utilized in analyses of the SBR cohort with consideration of the following model parameters: cohort size; types of leukemia assessed; BD exposure variables included; whether lagged or transformed exposure variables were applied; and which nonexposure covariate variables were included. After their thorough impact analysis, the authors recommended that subsequent risk assessment efforts rely on β-coefficients produced from analyses of the latest and largest update of the SBR cohort, derived from Cox regression models for the dose-response relationship between non-lagged and non-transformed cumulative BD exposure (parts per million-years – ppm-years) and leukemia mortality, and adjusted for age and peak exposure to BD (i.e., tasks with exposures ≥100 ppm, referred to as BD-high intensity tasks [BD-HITs]). To date, an analysis following the totality of these specific recommendations has not been conducted. Similarly, an independent science advisory panel convened for the recent Kirman et al. [15] study largely agreed on analyzing the most current update of the SBR data using a Cox proportional hazard model adjusted only for statistically significant covariates as a basis for human health risk assessment.

Moreover, recent analyses of the SBR cohort reveal limitations and inconsistencies in the SBR data. As described by Sathiakumar et al. [8], inclusion criterion for males in the SBR cohort was restricted to those who had worked for ≥1 year, whereas criterion for female workers allowed for only 1 day of employment for inclusion. Additionally, Sathiakumar et al. [8,10] described that data on work histories and BD exposure were no longer collected or estimated after 1991. Although some workers who were actively employed at the end of 1991 still had some level of BD exposure, Sathiakumar et al. [8,10] included these workers without making attempts to update their exposure histories beyond 1991. Sathiakumar et al. [10] reported that, among workers from 8 plants, 4243 workers were still employed at the end of 1991, while Sathiakumar et al. [8] reported that, among workers from the current SBR cohort (i.e., 6 plants), 4079 were still employed after exposure assessment terminated. Finally, Sathiakumar et al. [10] updated vital status of the SBR cohort in 2009 with high linkage rates; in their subsequent analysis of workers from 6 plants, Sathiakumar et al. [8] noted that 99% of the 21 087 workers in the cohort had updated vital status information. Thus, their analysis contained the full cohort of 21 087 workers.

Therefore, the objective of this study was to perform an analysis of the most recent SBR cohort data (i.e., the 2009 update). The current analysis expands upon previous analyses of the same data in that it followed the recommendations of Antoniou and Kirman [14], and included additional explorations of covariates and worker inclusion criteria to evaluate the quantitative effects on the ERR between BD and leukemia mortality. Specifically, the authors assessed the effect of model adjustments and exclusion criteria on the observed relationship between cumulative BD exposure (in ppm-years) and all leukemia mortality by comparing β-estimates and standard errors (SE) across models.

## MATERIAL AND METHODS

Four deidentified datasets, with a common identifier for linkage, were obtained from the University of Alabama at Birmingham (UAB), USA. Specifically, the authors received a male worker file (N = 16 579), a female worker file (N = 4509), a worker-by-observation-day file (N = 292 621 832), and a worker-by-job-specific file (N = 386 838). Due to data sharing restrictions of the Centers for Medicare and Medicaid Services (CMS), 19 deceased females included in the female worker file whose vital status came from CMS were excluded from the worker-by-observation-day file. Overall, these data contained demographic, SBR work history, SBR occupational exposures (BD and STY estimates only), and vital status of 21 069 male and female workers from 6 North American SBR plants – 1 in Kentucky, 1 in Ontario, and 2 each in Louisiana and Texas. Demographic covariates provided in the data included age, race, and sex. Work history variables included year of hire, plant, duration of employment, and employment type (ever hourly, yes/no).

The data files described above contained various exposure and outcome variables that were developed previously by the UAB researchers. The present analysis utilized these previously developed exposure and outcome variables, such that no new exposures were estimated, and no new mortality outcomes were assessed. As described in Macaluso et al. [16], BD and STY exposures were characterized from 1944 through 1991. Briefly, job-, task-, year-, and plant-specific exposure estimation models were developed through plant inspection, record review, and interview of key personnel by industrial hygienists and chemical engineers. Worker histories were used to estimate exposures for each worker in the cohort. Cumulative BD and STY exposure were provided in the worker-by-observation-day file.

Vital status was ascertained from 1943 through 2009, as described in Sathiakumar et al. [10]. Participants were followed until death, loss to follow-up, or the end of the study (i.e., 2009). International Classification of Diseases (ICD) codes for deceased workers were obtained from the National Death Index (NDI) and the Canadian Mortality Database (CMDB) for USA and Canadian employees, respectively. Codes provided for underlying cause of death or ≤5 contributing causes of death were used to confirm all leukemia mortality (ICD-9 codes, 204–208; ICD-10 codes, C91–C95).

Descriptive statistics were conducted with a dataset created from the last observation per worker (i.e., the highest cumulative exposure) from the worker-by-observation-day dataset. The authors analyzed sex, plant, employment type, race, year of hire (grouped by quartile), duration of employment, and BD-HITs, by leukemia mortality and by BD exposure. Relationships between the variables and leukemia and BD exposure were evaluated for statistical significance using chi-square or Wilcoxon tests, respectively. The authors also assessed mean (M) ± standard deviation (SD), median (Me), interquartile range (IQR), minimum (min.), and maximum (max) of age at hire, age at end of follow-up, and cumulative STY and BD exposure for the cohort. Descriptions of workers with unknown vital status (N = 242), women with <1 year of employment (N = 1801; all men in the dataset had ≥1 year of employment), and workers employed at the end of 1991 (N = 3723) were included in descriptive statistics.

To evaluate the association between BD and leukemia mortality, the authors used Cox proportional hazards regression to estimate adjusted β-estimates, SE, hazard ratios (HRs), and 95% CIs. In all Cox models, the time scale represented the number of days from hire date to each person-day observation, and cumulative BD was included as a time-varying exposure. The authors ran the following Cox regression models to assess the effect of model adjustments and exclusion criteria on the observed relationship between BD exposure (in ppm-years) and all leukemia mortality:

– model 1: As recommended by Antoniou and Kirman [14], this model includes cumulative BD exposure and 2 covariates (age at day of observation and total BD-HITs). Counts of BD-HITs were only provided in the worker-by-job-group dataset. Therefore, BD-HITs were linked to the worker-by-observation-day dataset as a sum across all jobs for each worker and could not be evaluated as a time-varying covariate.
– model 2: This model added to model 1, any covariate found to be statistically significantly (at p < 0.05) associated with BD exposure and leukemia mortality, as recommended by Kirman et al. [15]. The Akaike information criterion (AIC) of this model was compared to model 1. All subsequent models were adjusted with the same covariates as the model with a lower AIC (i.e., either model 1 or model 2).

As indicated above, models 3–6 excluded specific populations in order to investigate the stability of β and SE estimates applying different exclusion criteria:

– model 3: This model excluded workers with unknown vital status (N = 242),
– model 4: This model applied consistent duration of employment criteria between males and females (i.e., excluded women with <1 year employment, N = 1801),
– model 5: This model excluded workers who were still employed at the end of 1991. An indicator for workers employed at the end of 1991 was not included in the UAB shared datasets. Therefore, a variable was created for this purpose and was calculated as the sum of duration of employment and year of hire. A conservative estimate for this population (N = 3723) was determined by applying a cut-off of ≥1991 based on this new variable. However, this estimate was considerably lower than the count provided in Sathiakumar et al. [8] (i.e., 4079),
– model 6: This model excluded all worker groups described in model 3, model 4, and model 5 (i.e., all above exclusions applied concurrently).

Kirman et al. [15] noted that although testing the proportional hazards assumption is “good practice”, it has either not been conducted or has not been included in prior publications of the SBR data. Therefore, the authors plotted and inspected Schoenfeld residuals to test the proportionality of the hazards assumption. The authors examined Martingale residuals to assess the assumption of the linear relationship between the exposure and outcome variables on the log-hazard scale, and the authors performed a collinearity analysis to assess multi-collinearity of covariates. These tests were all conducted including variables as in either model 1 or 2 (the model with the lower AIC) and after exclusion groups were explored. SAS software, v. 9.4 (SAS Institute Inc., Cary, NC, USA) was used for all analyses.

## RESULTS

Descriptive results of the 21 069 workers included in the current analysis can be found in Table 1 and 2. The workers in the cohort were mostly male (78.7%) and white (88.1%). The M±SD age at hire and end of follow-up was 28.8±8.7 years and 68.0±12.9 years, respectively. About a third of the cohort worked in the Ontario, Canada plant (33.4%) and only 28.3% of the workers were never hourly-workers. While 41.2% of the cohort had <5 years of employment in the SBR facilities, 19.9% of them were employed for ≥25 years. In total, there were 132 (1.4%) deaths from leukemia. Regarding exposure, 66.4% of the cohort was exposed to BD and 73.2% were exposed to STY. Among the workers who were exposed to BD, most had BD-HITs (89.9%). Sex, race, plant location, ever hourly status, year of hire, duration of employment, and STY exposure were all statistically significantly associated with BD exposure. However, only year of hire was also statistically significantly associated with leukemia mortality.

**Table 1.** Age and select exposure characteristics of members of the North American styrene-butadiene rubber cohort, 1943–2009

Variable	Me (IQR)	M±SD	Min.–max	Participants (N = 21 069) [n (%)]
Age [years]				
at hire	26 (22–34)	28.8±8.7	12–67	21 069 (100)
at end of follow-up	69 (59–78)	68.0±12.9	18–110	21 069 (100)
Exposure [ppm-years]				
butadiene	48.1 (10.6–167.6)	187.0±517.6	>0.00–9264.0	13 999 (66.4)
styrene	11.3 (2.8–36.0)	38.1±97.7	>0.00–1618.3	15 415 (73.2)
Butadiene HITs	612.3 (153.9–2077.2)	1997.8±3976.5	0.04–55 365.4	12 584 (59.7)

HITs – high-intensity tasks; IQR – interquartile range; ppm-years – parts per million-years.

**Table 2.** Demographic and employment characteristics of members of the North American styrene-butadiene rubber cohort by 1,3-butadiene (BD) exposure and leukemia mortality outcome, 1943–2009

Variable	Participants (N = 21 069)		BD exposure [ppm-years] (Me (IQR))	p^a^	Deaths [n (%)]		p^b^
	total [n (% of total)]	BD exposed [n (%)]			total	due to leukemia	
Total cohort		13 999 (66.4)	48.1 (10.6–167.6)		9647 (45.8)	132 (1.4)	
Demographic characteristics							
sex				<0.0001			0.3740
male	16 579 (78.7)	12 814 (77.3)	53.9 (13.0–177.7)		8214 (49.5)	116 (1.4)	
female	4490 (21.3)	1185 (26.4)	8.1 (1.6–45.8)		1433 (31.9)	16 (1.1)	
race				<0.0001			0.9586
white	18 565 (88.1)	12 211 (65.8)	44.1 (10.0–149.0)		8419 (45.4)	115 (1.4)	
non-white^c^	2504 (11.9)	1788 (71.4)	97.5 (16.0–343.9)		1228 (49.0)	17 (1.4)	
Employment characteristics							
plant location				<0.0001			0.6562
Kentucky	1563 (7.4)	1144 (73.2)	75.3 (14.6–244.8)		764 (48.9)	13 (1.7)	
Louisiana	2462 (11.7)	1815 (73.7)	72.2 (19.7–229.8)		1180 (47.9)	13 (1.1)	
Louisiana	2839 (13.5)	1520 (53.5)	73.4 (15.0–320.3)		1454 (51.2)	15 (1.0)	
Texas	2925 (13.9)	1689 (57.7)	38.1 (10.0–133.7)		1473 (50.4)	19 (1.3)	
Ontario	7044 (33.4)	4936 (70.1)	43.2 (8.3–143.5)		2652 (37.7)	39 (1.5)	
Texas	4236 (20.1)	2895 (68.3)	31.6 (8.6–119.8)		2124 (50.1)	33 (1.6)	
ever hourly				<0.0001			0.7466
no	5972 (28.3)	2127 (35.6)	7.4 (1.6–22.7)		1935 (32.4)	25 (1.3)	
yes	15 097 (71.7)	11 872 (78.6)	64.8 (16.3–203.4)		7712 (51.1)	107 (1.4)	
hire year				<0.0001			0.0007
quartiles							
1939–1949	5388 (25.6)	3596 (66.7)	77.0 (20.2–253.5)		4552 (84.5)	41 (0.9)	
1950–1959	5611 (26.6)	4004 (71.4)	97.4 (29.6–265.8)		3325 (59.3)	66 (2.0)	
1960–1971	4980 (23.6)	3250 (65.3)	39.6 (10.1–129.2)		1292 (25.9)	17 (1.3)	
1972–1990	5090 (24.2)	3149 (61.9)	10.9 (2.8–37.0)		478 (9.4)	8 (1.7)	
dichotomized at 1960				<0.0001			0.8596
before 1960	10 999 (52.2)	7600 (69.1)	87.2 (24.5–260.5)		7877 (71.6)	107 (1.4)	
1960 and later	10 070 (47.8)	6399 (63.5)	20.2 (5.0–75.0)		1770 (17.6)	25 (1.4)	
duration of employment				<0.0001			0.4107
<5 years	8681 (41.2)	4292 (49.4)	12.9 (3.6–36.4)		3300 (38.0)	37 (1.1)	
5 to <15 years	4848 (23.0)	3320 (68.5)	40.3 (10.7–119.8)		1815 (37.4)	26 (1.4)	
15 to <25 years	3347 (15.9)	2718 (81.2)	90.2 (26.5–228.8)		1959 (58.5)	27 (1.4)	
≥25 years	4193 (19.9)	3669 (87.5)	162.9 (57.7–366.1)		2573 (61.4)	42 (1.6)	
styrene exposed				<0.0001			0.1578
no	5654 (26.8)	346 (6.1)	1.0 (0.3–21.5)		2173 (38.4)	23 (1.1)	
yes	15 415 (73.2)	13 653 (88.6)	50.3 (11.4–169.8)		7474 (48.5)	109 (1.5)	
butadiene HITs				<0.0001			<0.0001
low (≤Me)	14 777 (70.1)	7707 (52.2)	14.8 (3.9–48.8)		5987 (40.5)	58 (1.0)	
high (>Me)	6292 (29.9)	6292 (100)	137.3 (59.3–335.3)		3660 (58.2)	74 (2.0)	
excluded populations							
workers with <1 year employment	1801 (8.6)	374 (20.8)	2.1 (0.6–7.6)	n.a.	721 (40.0)	9 (1.3)	n.a.
employed after 1991	3723 (17.7)	2797 (75.1)	35.3 (8.0–133.9)		437 (11.8)	9 (2.1)	
unknown vital status	242 (1.2)	112 (50.4)	16.8 (8.2–61.5)		unknown	unknown	

HITs – high-intensity tasks; IQR – interquartile range; n.a. – not applicable; ppm-years – parts per million-years.

a Wilcoxon.

b Chi^2^.

c Non-white group includes Asian, Black, Hispanic, American Indian, other, and unknown racial/ethnic categories.

When comparing models 1 and 2, model 2 – adjusted for age, BD-HITs, and year of hire – had a lower AIC than model 1, which was not adjusted for year of hire (Table 3). In Cox proportional hazards models conducted with the full cohort and adjusted for age, BD-HITs, and year of hire (model 2), the risk of leukemia mortality increased by approx. 0.022% (95% CI: 0.005–0.039%) for every ppm-year of BD exposure (Table 3).

**Table 3.** Comparison of Cox regression model parameters for 1,3-butadiene (BD) between models 1 and 2, full North American styrene-butadiene rubber cohort (N = 21 069, person-years = 801 212.6), 1943–2009

Model	β	95% CI	SE	HR	95% CI	p	AIC
Model 1	2.38 × 10^–4^	0.74–4.01 × 10^–4^	8.36 × 10^–5^	1.000238	1.000074–1.000402	0.0045	2361.29
Model 2	2.21 × 10^–4^	0.49–3.93 × 10^–4^	8.78 × 10^–5^	1.000221	1.000049–1.000393	0.0118	2354.56

AIC – Akaike information criterion; HITs – high-intensity tasks; HR – hazard ratio; SE – standard error.

Model 1 – adjusted for age and BD-HITs; model 2 – adjusted for age, BD-HITs, and year of hire.

Table 4 presents the results for models 3–6, adjusted for the model 2 covariates (age, BD_HITs, and year of hire). Excluding all workers with <1 year of employment decreased β and increased the SE (model 4: β = 2.14 × 10^–4^, 95% CI: 0.40–3.87 × 10^–4^, SE = 8.84 × 10^–5^, HR = 1.000214, 95% CI: 1.000040–1.000387), excluding workers employed at the end of 1991 and applying all exclusion criteria concurrently increased both β and the SE (model 5: β = 2.31 × 10^–4^, 95% CI: 0.52–4.11 × 10^–4^, SE = 9.14 × 10^–5^, HR = 1.000231, 95% CI: 1.000052–1.000411 and model 6, β = 2.23 × 10^–4^, 95% CI: 0.41–4.04 × 10^–4^, SE = 9.25 × 10^–5^, HR = 1.000223, 95% CI: 1.000041–1.000404), while excluding workers with unknown vital status (model 3) had no effect on β or SE.

**Table 4.** Comparison of Cox regression model parameters for 1,3-butadiene (BD) when excluding specific populations of the North American styrene-butadiene rubber cohort, 1943–2009

Model	Employees included [n]	Person-years of included employees	Leukemia deaths [n]	β	95% CI	SE	HR	95% CI	p
Model 3	20 827	800 795.1	132	2.21 × 10^–4^	0.49–3.93 × 10^–4^	8.78 × 10^–5^	1.000221	1.000049–1.000393	0.0118
Model 4	19 268	723 846.8	123	2.14 × 10^–4^	0.40–3.87 × 10^–4^	8.84 × 10^–5^	1.000214	1.000040–1.000387	0.0157
Model 5	17 346	676 760.1	123	2.31 × 10^–4^	0.52–4.11 × 10^–4^	9.14 × 10^–5^	1.000231	1.000052–1.000411	0.0114
Model 6	15 387	598 997.7	114	2.23 × 10^–4^	0.41–4.04× 10^–4^	9.25 × 10^–5^	1.000223	1.000041–1.000404	0.0160

HR – hazard ratio; SE – standard error.

Model 3 – excluded workers with unknown vital status at the end of follow-up; model 4 – excluded workers with <1 year of employment; model 5 – excluded workers employed at the end of 1991; model 6 – excluded all groups defined in models 3–5.

## DISCUSSION

This manuscript reports on an analysis of the latest update of the SBR cohort using Cox proportional hazards models with number of days from hire date to person-day observation as the time scale and including cumulative BD as a time-varying exposure. Although the authors first ran a model adjusted for age and BD-HITs (model 1), as recommended by Antoniou and Kirman [14], the authors determined that the model including year of hire (model 2), the only additional statistically significant covariate associated with BD and leukemia in descriptive analysis, was a better fit when comparing AIC values from both models. Additionally, excluding female workers with <1 year of employment (model 4) aligned female inclusion criteria to male worker criteria applied in all prior analyses of the SBR cohort. Although excluding workers employed at the end of 1991 is appropriate due to potential exposure misclassification, the inability to precisely identify this group in the authors' data reduces the authors' confidence in this analysis. Therefore, the authors propose the model which excluded female workers with <1 year of employment (N = 1801) and adjusted for age, BD-HITs, and quartile of hire year as the preferred model for quantitative assessments of risk (model 4, β = 2.14 × 10^–4^, 95% CI: 0.40–3.87 × 10^–4^, HR = 1.000214, 95% CI: 1.000040–1.000387).

Although 4 analyses were previously conducted with the latest and largest update of the SBR study [8–11], this is the first analysis, to the authors' knowledge, to carefully consider and incorporate the recent recommendations for modeling the ERR for BD exposure and leukemia mortality [14,15]. Specifically, the authors' analysis adhered to all the recommendations for the selection of outcome, exposure, and adjustment variables put forth by Antoniou and Kirman [14]. However, the study also followed the statistical recommendation made by the expert panel assembled by Kirman et al. [15], including only covariates that were statistically significant in the model. While many of these recommendations were incorporated into the recent BD-leukemia analyses that utilized up-to-date SBR data through 2009 (e.g., utilizing Cox regression, using non-lagged and non-transformed data, including age as a covariate), the main difference between the authors' analysis and the others surrounds the selection of covariates for the models. Most notably, the difference pertains to whether BD-HITs was considered. When comparing the authors' findings with previous studies, it is important to note that the study population differed slightly from that used in the latest update of the SBR data because of the CMS data-sharing restrictions described above.

Nevertheless, the results were largely consistent with recent assessments, although comparison with Sathiakumar et al. [10] was not possible because β-coefficients from their regression models were not reported. Sathiakumar et al. [8] reported a β = 2.55 × 10^–4^ with a 95% CI: 0.52–4.57 × 10^–4^ from their Cox regression analysis, which used age at each day of follow-up as the timescale and adjusted for age at hire, year of hire, race, sex, plant, and ever hourly status. Sathiakumar et al. [9] reported a β = 2.9 × 10^–4^ with a 95% CI: 0.9–4.9 × 10^–4^ from their multivariate model that adjusted for age, race, year of birth, and plant and used age as the time scale. Valdez-Flores et al. [11] reported a β = 2.81 × 10^−4^ with a 95% CI: 1.17–4.45 × 10^–4^ from their univariate Cox regression analysis, which used age as the timescale. However, when the researchers adjusted for BD-HITs, the β-estimate for BD was reduced by >50% and was no longer statistically significant (β = 1.32 × 10^–4^, 95% CI: –0.80–3.43 × 10^–4^). The reason for the attenuation observed by Valdez-Flores et al. [11] is unclear but may have been due to their reliance on the worker data for their Cox models versus the worker-by-observation-day data that were utilized in the current analysis. In their methods, the authors explained that UAB provided them with exposure histories at the worker-level (i.e., total cumulative exposure to BD rather than time-varying cumulative BD), and that they used the exposure history of each worker to fit their Cox models, thus, it appears that the research team only had access to the worker files. Moreover, there was no mention of time-dependent variables nor was person-time reported, which are likely further indication that the models were not fit with person-day data. Thus, this considerable relative change may indicate that the sum of BD-HITs and total cumulative exposure to BD are more correlated than the sum of BD-HITs and time-varying cumulative BD used in the authors' models.

In an earlier analysis of the SBR cohort, Cheng et al. [4] conducted a thorough examination of the influence of BD-HITs on the BD-leukemia relationship that included independent examinations of number of BD-HITs and average intensity of BD exposure. However, they also ran a Cox regression model that adjusted for both cumulative BD and BD-HITs as well as age, and statistically significant β-coefficients were observed for both exposure metrics (β = 2.5 × 10^–4^, p = 0.03 and β = 5.1 × 10^–5^, p = 0.01, respectively). The estimate for BD-HITs in the authors' model was slightly lower than that observed by Cheng et al. [4] but also statistically significant (β = 3.4 × 10^–5^, p < 0.01, data not previously shown). While peak BD exposures are captured in the cumulative BD measure, chronic exposure and intense exposure to BD may not carry the same biological risk [17–19]. The fact that BD-HITs is statistically significant in the authors' model is likely an indication that the risk associated with cumulative BD exposure is partially attributable to BD-HITs.

Although the authors did not conduct a formal, directly comparable cancer risk assessment using methods aligned with the ECHA RAC approach, it is helpful to contextualize their findings within the framework of occupational risk. According to the life-table analysis conducted by ECHA, “the lifetime risk of dying from all leukaemia (background mortality, 0–85 years) in the EU population (2012–2017) was 0.57% (5.7/1000, 57/10 000 or 570/100 000)” [13]. Applying the findings from model 4 – which excluded all workers with <1 year of employment and adjusted for age, BD-HITs, and year of hire – to this overall leukemia mortality background in the EU (and assuming a 40-year work duration), the authors estimate a BD exposure of 0.82 ppm to result in an excess of 4 leukemia deaths per 100 000 workers. Using the 95% upper bound of the authors' β-estimate for this calculation (i.e., 3.87 × 10^–4^), a BD exposure of 0.45 ppm is expected to result in an excess of 4 leukemia deaths per 100 000 workers. This exposure based on the conservative upper bound estimate is greater than the ECHA exposure estimate of 0.065 ppm and the DECOS exposure estimate of 0.047 ppm for the same excess life-time cancer risk [13,20]. Although a small portion (<5%) of the difference between the ECHA exposure estimate and the authors' exposure estimate is due to not calculating excess risk by age category in the authors' analysis, most of the difference results from modelling on grouped data vs. modelling on individual-level data and differing model assumptions (i.e., linear vs. log-linear).

The strengths of the authors' study include access to daily, individual-level data of the large SBR cohort with a detailed and robust exposure assessment conducted by Macaluso et al. [16], Cox proportional hazards analyses which allow exposure and age to be analyzed continuously, and a streamlined analysis starting with a model suggested by Antoniou and Kirman [14]. Additionally, the authors verified both the proportional-hazards assumption and the linearity of the exposure-outcome relationship on the log-hazard scale (Figure 1 and 2). Although analyses on the SBR dataset have been relatively consistent over various exposure assessment and mortality updates [14], an update of mortality in the cohort – last assessed in 2009 – would be useful, especially to investigate the effect of reductions in BD exposure in the cohort after the 1950s [8]. The relatively few deaths in the cohort among workers hired in 1960 or later (17.6% compared to 71.6% in those hired before 1960) make the current data uninformative on such effects. Additionally, indirect adjustment methods should be explored to adjust for potential confounding factors, including smoking. There is limited knowledge of additional risk factors among the SBR cohort, including smoking, but these unmeasured factors may influence the observed BD ERR.

**Figure 1.**
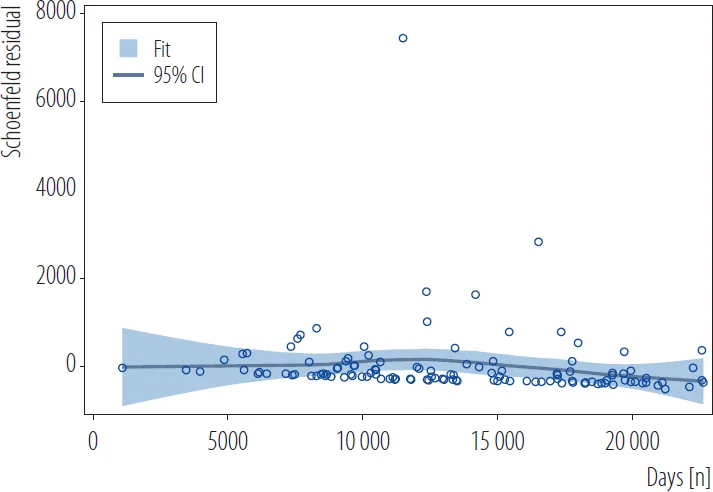
Schoenfeld residuals of cumulative 1,3-butadiene (BD) exposure vs. time in members of the North American styrene-butadiene rubber cohort, 1943–2009

Days from hire date to date of person day observation.

**Figure 2.**
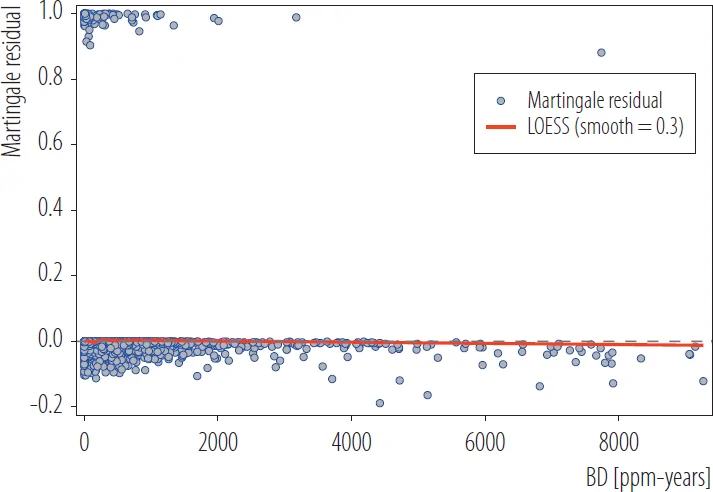
Martingale residuals vs. cumulative 1,3-butadiene (BD) exposure in members of the North American styrene-butadiene rubber cohort, 1943–2009

LOESS – locally estimated scatterplot smoothing.

## CONCLUSIONS

The authors' exploration of covariates and worker inclusion criteria to evaluate the quantitative effects on the ERR between BD exposure and leukemia mortality demonstrates that the relationship has little variation despite covariates and worker groups included. Using the 95% upper bound of the β-estimate from the authors' preferred model to estimate excess risk results in a BD exposure greater than both the ECHA and DECOS estimates for the same excess life-time cancer risk, suggesting a weaker overall ERR.
